# Older Brazilian women’s experience of psychological domestic violence: a social phenomenological study

**DOI:** 10.1186/s12939-015-0173-z

**Published:** 2015-05-12

**Authors:** Rafaella Queiroga Souto, Miriam Aparecida Barbosa Merighi, Sepali Guruge, Maria Cristina Pinto de Jesus

**Affiliations:** RN PhD candidate, University of Sao Paulo, Nursing, 419 Dr. Eneas Carvalho de Aguiar Av. 05403, Sao Paulo, 05403-000. Brazil; Vice-director and professor, University of Sao Paulo, Nursing, 419 Dr. Eneas Carvalho de Aguiar Av. 05403, Sao Paulo, 05403-000 Brazil; Associate Professor, Ryerson University, School of Nursing, 350 Victoria Street, Toronto, ON M5B2K3 Canada; Visiting professor, University of Sao Paulo, Nursing, 419 Dr. Eneas Carvalho de Aguiar Av. 05403, Sao Paulo, 05403-000 Brazil

**Keywords:** Domestic violence, Elder abuse, Older people, Psychological violence, Qualitative approaches, Social Phenomenology, Violence, Women’s health

## Abstract

**Background:**

Domestic violence is a global public health issue, as it is in Brazil. The psychological violence is one of the most prevalent forms of domestic violence, affecting more women than men. However, many older adults do not consider it as a type of domestic violence. In addiction, psychological violence has received little attention from researchers. So, this study aims to further understand the phenomenon of psychological domestic violence perpetrated by relatives against older adult women (60 years and older).

**Methods:**

A qualitative study was conducted using a social phenomenological approach proposed by Alfred Schütz. In-depth interviews were conducted with 11 older Brazilian women from three different agencies, two in Campina Grande and one in São Bernardo do Campo. Data collection took place between November 2012 and February 2013. We performed data analysis using the key concepts (such as the world of everyday life, natural attitude, intersubjetivity, stock of knowledge, biographical situation, social action, motivation and typification) proposed by Alfred Schutz.

**Results:**

Despite the fact that participants reported being psychologically abused, they also referred to being neglected and financially abused. They revealed being threatened, disrespected, neglected, financially abused, forced to do housework, and humiliated. Older women expressed feelings of sadness, anger, grief, and fear, which had negative effects on their health. Attempts by the participants to change their current situations were unsuccessful and resulted in feelings of helplessness. The abuser’s behaviour will change, and leaving the abusive situation were two possible outcomes pointed for participants.

**Conclusions:**

A support network is crucial to help changing the behaviour of aggressors and/or to help older adult women leave the abusive situation. Further research is needed to understand the risk factors linked to abuse behaviours, to develop educational programs for the abusers, and to design social support for the victims.

**Electronic supplementary material:**

The online version of this article (doi:10.1186/s12939-015-0173-z) contains supplementary material, which is available to authorized users.

## Background

Violence against older adults is a complex and multidimensional problem that impacts on social, psychological and physical dimensions, affecting the public heath and the human rights [1]. According to the International Network for the Prevention of Elder Abuse (INPEA), violence against older adults is defined as: “a single, or repeated act, or lack of appropriate action, occurring within any relationship where there is an expectation of trust, which causes harm or distress to an older person” [2].

The World Health Organization (WHO) states that the prevalence of violence experienced by the elderly ranges from 1% to 35% [3]. In Brazil, two studies [4, 5] have analysed the prevalence of physical violence against older men and women in small towns: Camaragibe, Pernambuco [6] with 144,466 inhabitants [4]; and Niterói, Rio de Janeiro [7] with a population of 487,562 [4]. These studies (with sample sizes of 315 and 343 subjects respectively) reported that the prevalence of physical domestic violence against older adults was about 10% [6–7]. This results indicate that the prevalence found in these Brazilian studies is high when compared with 2.2% in Ireland [8] and 2.6% in United Kingdom [9]. On other hand, the findings of Brazilian studies is close to those found in Canada, which range between 4% and 10% [2], in Spain (11.9%) [9], and in Portugal (12.3%) [10]. However, they are low when compared with 18.4% in Israel, with 21.4% in Hong Kong [9] and a range from 13.9% to 25.8% in Chinese older adults living in the Greater Chicago area [11].

Among the subtypes of violence against older adults, psychological abuse is one of the most prevalent [12]. Results of a study on psychological abuse among older women conducted in the United States of America indicate a prevalence of 45%. Among those who have suffered psychological abuse, 48% reported two or more abusive actions, such as: abusive language, overly critical, screaming, possessive behaviour or jealousy signs. Furthermore, among older women experiencing different subtypes of violence, researchers found a range of 86% and 95% suffering psychological abuse [12]. Among Portuguese people, the prevalence of psychological abuse was 6.3%, the same as financial (6.3%). Other types of violence included: Physical abuse (2.3%), neglect (0.4%), and sexual abuse (0.2%) [10]. The prevalence of psychological abuse among Chinese older adults living in the Greater area of Chicago was 1.1%-9.8%, physical abuse was 1.1%, sexual abuse was 0.2%, caregiver neglect was 4.6%-11.1%, and financial exploitation was 8.8%-9.3% [11].

The psychological abuse may occur in isolation or in combination with other kinds of abuse [12]. However, many older adults do not consider it as a type of domestic violence [13]. In addiction, psychological abuse tends to receive little attention from researchers [14].

Psychological abuse of older adults refers to “a verbal or gestural action whose aim is to terrorize, to humiliate, to restrict freedom, or to isolate older adults from society” [15]. This subtype of abuse can bring negative consequences to the victims’ life and health, what includes decreased sense of power, shame, fear of retaliation, fear of institutionalization, isolation, anxiety, depressive symptoms and changes in health status [14].

The other types of abuse are defined as: physical abuse, financial/material abuse, sexual abuse, and neglect. First is defined as the infliction of pain or injury, physical coercion, or physical/chemical restraint. Second is described as the illegal or improper exploitation and/or use of funds or resources. Third corresponds to a non-consensual contact of any kind with an older person and forth fourth is the intentional or unintentional refusal or failure to fulfil a care-taking obligation’ [2,3].

Both men and women can be categorized as either perpetrators or victims of domestic violence. However, more than 70% of the victims of all types of violence are women [16]. In surveys, women who reported experiencing psychological violence also reported a lower quality of health than those experiencing physical violence [14].

INPEA highlighted that whereas one in four older adults are at risk of domestic violence, only a small proportion of domestic violence against older adults is actually reported [2]. Underreporting may be due to: victims hesitating to admit they are being victimized, religious and cultural beliefs, social norms, fear of reprisal, protection provided for the abuser, shame, guilt, helplessness, and despair [17]. In Brazil, the report of abuse of older adults is mandatory for health professionals such as nurses, physicians, dentists, and psychologists [18]. However, a study found that 60% of doctors and nurses in Brazil have not received any formal education about domestic violence and, consequently, they feel unprepared to provide appropriate care and referral [19]. The most cases of domestic violence against older adults in Brazil are only reported when they result in physical injuries [20].

Brazil currently has 23.5 million people over 60 years old, representing 12.1% of the total population from which 13.1 million (55.70%) are women [4]. The number o folder adults expected in 2025 is approximately 30 million, what is equivalent to 15% of the population [5]. Analysing social and economic aspects of the Brazilian population, we can state that illiteracy decreased, from 11.5% in 2004 to 8.3% in 2013; and schooling increased from 78.1% in 2012 to 81.2% in 2013. Regarding to the poverty, about one million people left misery in 2012 and the income inequity lowered by 54.9% from 2002 to 2012. However, in Brazil, there still are 15.7 million people living in poverty and a high level of social inequity [4]. This context stems from the relationship of command/subservience among poor people and the owners of farms, a structure that formed the basis of the Brazilian culture, even after the abolishment of slavery.

Moreover, Brazilian culture is marked by traditional gender values and patriarchy, in which the male is defined as the subject of sexuality and the female as its object [21]. Brazilians have often considered themselves as a nonracist society due to the historical blending of indigenous American, Iberian, and African peoples into a single national identity. However, ‘race’ in Brazil is embodied in everyday valuations of sexual attractiveness that are gender, race, and class-oriented. Ageism in Brazil should be addressed in the context of multiple discriminations [22].

In response to the international movement for adopting specific measures to protect older adults, the Brazilian National Elderly Policy was published in 1994 [23]. The Senior Citizens’ Statute was published in 2003 [24], and the National Elderly Health Policy was published in 2006 [25]. These policies and documents have helped improve legal protections for older adults and have strengthened legal guidelines to address violent situations among this population. Despite such programs, statistical trends of psychological domestic violence against older people in Brazil are still not known.

There is also a lack of qualitative research focused on psychological domestic violence from the older women’s point of view. Therefore, the aim of this study is to understand older adult women’s experience of psychological domestic violence. The primary research question for this study was: What are older Brazilian women’s experiences of psychological domestic violence? The related questions were: 1) How older Brazilian women experience their daily life when they are victims of psychological domestic violence? 2) How do older Brazilian women respond to psychological domestic violence? and 3) What are older Brazilian women’s needs, expectations, and aims in dealing with the psychological domestic violence in their lives?

## Methods

A qualitative study was conducted using a social phenomenological approach that was designed to help clarify the social relations underlying the domestic violence [26]. In particular, we applied Alfred Schutz’s work [27], which allows the investigation of individual behaviour, and the understanding of the experiences lived by a social group in a specific situation. We explored the Schutz’s key concepts that are related to older adult women subjected to psychological violence. We used his concepts throughout the paper as our lens for data analysis and reporting. These concepts were: the world of everyday life, natural attitudes, intersubjectivity, stock of knowledge, biographical situation, social action, motivation (reason why and reason for), and typification [27]. According to Schutz, interpreting human action requires identifying individuals’ ‘world of everyday life’ [28]. Within the ‘world of everyday life’, people act in a natural way, based on what is presented to them as a social reality. The ability to intervene in this world, influencing and being influenced, is called ‘natural attitude’ [28]. ‘Intersubjectivity’ is a precondition of social life: the source of meanings. Experiences are highly individualized and stem from the meanings that each individual attaches through consciousness. So, the experiences of the phenomenon become visible through individuals reflection. Her/his own interests, motives, desires, and ideological and religious commitments shape these interpretations, and her/his experiences bring a ‘stock of knowledge’ that is available according to the ‘biographical situation’ of each person. Therefore, the individual’s choice for an action is always related to other actions, to the current context, and to the future behaviours. Whether manifest or latent, the chosen action becomes a social reality and presupposes a typification, what means that an individual will act typically the same way in a similar situation. Individuals choose an action based on their motives/motivations: ‘reasons why’ and ‘reasons for’ [27]. A ‘reason why’ is related to past experiences and is an objective category accessible for a researcher. A ‘reason for,’ which guides future actions, is subjective. Researchers may capture an individual’s experience through the identification of his/her ‘reason for’ (an anticipated or imagined action) in relation to the experienced situation. Schutz’s theory was applied throughout the research process to ensure the methodology consistency [29].

### Context of the study site

This research involved one social agency in São Bernardo do Campo, São Paulo, in the South-East of Brazil and two agencies (one social and one legal) in Campina Grande, Paraiba, in the North-East Brazil. Due to the difficulty of recruiting participants, one service was not enough for this study. Thus, three agencies were needed to obtain a representative sample to answer all the research questions. In this sense, we did not select a forth agency. It was not necessary to select a fourth agency.

One of the social agencies is an older adult community centre, which promotes the social inclusion of older people and receives people with different needs. The second social agency is responsible for all older adult services in Campina Grande. One of these services is a free call service for reporting violence against older adult people. The legal service exclusively handles with cases involving domestic violence against women of all ages.

### Recruitment

Purposeful sampling was used to identify the participants. Recruitment began by presenting inclusion criteria to the leaders of each agency. These leaders were responsible for informing potential participants to the study. The inclusion criteria were: the participant must be registered at the agency as a victim of violence; be a woman; and be aged 60 years or over.

In São Bernardo do Campo, São Paulo, four older women were identified and invited to participate; three agreed. One potential participant declined because she did not want to talk about her own experience. The interviews were conducted in a private room located in one of the agencies, at a day and time suggested by the participants. In Campina Grande, Paraiba, a total of 28 possible participants were identified, but seven could not participate due to the lack of contact information. Consequently, 21 possible participants were contacted and invited to participate. Seven declined because they did not want to talk about this issue. Therefore, 14 interviews were conducted at a time and date that was convenient for each participant. Due to the participants’ requests, all interviews were conducted in their respective homes. Of these, six interviews were excluded because participants talked about domestic violence generally, rather than their individual experience. Therefore, the final sample for the data analysis consisted of 11 participants.

The study participants were aged from 66 to 85 years; they had an average of 5 children (range: 0–14 children), and 10 were widowed. Most participants had a low educational level: only two had completed higher education (university). Eight participants lived in their own home and the only source of income was the older adult retirement benefit. Two participants were living in their children’s home, and their retirement benefit was part of the entire family income. One participant had moved into a neighbour’s house and her income was the only household income.

About the family abusers, seven were female and six were male, being that four were daughters, two were granddaughters, four were sons, one was grandson, one was female caregiver and one was male caregiver. The number of aggressors was higher than the number of victims because there were cases in which the victim had more than one aggressor.

### Data collection

Data collection occurred between November 2012 and February 2013 and was comprised of open-ended interview questions (e.g. Tell me about your daily life related to the violence you have been subjected to?). Each interview lasted for one to two hours. They were audiotaped and, then, literal transcribed and translated to English. Interviews ended when each participant has said all she wanted about her experience on domestic violence.

The first author of this paper, who had been trained by her supervisor to conduct a phenomenological interview, asked open ended questions; respected participants’ wishes regarding the interview process, allowing participants to stop talking or to change the content of the interview as needed; provided information about counselling, legal and social services available in the participants’ cities and states; and gave to participants her personal contact information if they wanted to withdraw their participation from the study.

### Data analysis

Data analysis was conducted following the steps developed by social phenomenologists [26]. The participants’ speeches were read to determine the ‘meaning units’ that emerged from the ‘reasons why’ and from the ‘reasons for’ (Alfred Schutz’s concepts). The ‘meaning units’ were the words and phrases spoken by the women during the reporting of their experiences. Then, the first author grouped the ‘Meaning units’ to form themes, which is the name given by a researcher to summarize a group of ‘meaning units’. After generating these themes from the ‘meaning units’, which was captured by the first author using Alfred Schutz’s concepts, the first author assessed them together with the other authors to establish the ‘ideal/typical’ characteristics and experiences regarding to psychological domestic violence. Alfred Schutz’ motivational theory was used to understand and to discuss the findings as well. To ensure credibility and confirmability of the research, peer checking was used during the analysis. In this process, two of the authors separately conducted the initial data analysis, and the results were then compared. In cases of disagreement, one of the other authors was consulted to help take a decision about which themes best represented the participants’ experiences.

## Ethical considerations

The research ethics board at the University of São Paulo, and the three agencies granted approval for the study. Following this, participants were informed about the study and received the written consent forms. The signature of these forms was required to ensure the participation of individuals in the study. The information included the study objectives, the use of the audio-recorder, and how confidentiality would be ensured. Participants were advised that they were free to choose whether or not to participate in the study, that they could withdraw from the study anytime without any impact on their access to services, and that the collected data would be only used for the purpose of the study.

Confidentiality was maintained by: assigning all participants coded numbers to ensure data were anonymous; storing consent forms and identifiers in a locked cabinet (at the School of Nursing at the University of São Paulo); storing interview tapes in a separate locked cabinet (also at the School of Nursing); protecting all electronic information with an encrypted password; not sending any data by mail; and ensuring that leaders at each agency remained unaware of participants’ decisions to participate or not. None of the identifiers or raw data were available to anyone outside the research team. We destroyed interview tapes after the content were transcribed and checked for accuracy, and the transcripts will be destroyed five years after the completion of the study.

## Results

The findings are showed into two themes: ‘context of the violence’ and ‘expectations of the older women’. First category emerged from the participants’ ‘reason why’ and second one emerged from the participants’ ‘reason for’.

### Theme 1: Context of the violence

The context of the violence involves the participants’ daily life and actions . This theme is divided into three subthemes: manifestations of the violence; the impact of psychological violence on the participants; and the responses of the older women to violence.

#### Subtheme 1.1: Manifestations of the violence

The interviews revealed that the participants were threatened through verbal and non-verbal actions by the family members who used hurtful words and humiliating situations, and imposed their wishes upon the older women, disregarding the victims’ wishes and preferred habits. The excerpts below represent violent manifestations, including verbal abuse, threatening, lack of freedom, financial abuse, and isolation:

*She never hit me but she is bad with her words. Go away from here! Get out of here! She yells at me all the time. When I say anything to her, she breaks dishes, cups, everything. She has already thrown things on me! [Crying] She tells me: − If you say that to my sister, you will be in trouble. I cannot go anywhere. My sister always calls us to go visit her. But if I go I get scolded, so, no way! I do not go. (I#1)*

*My daughter talks to me just when she wants. When she wants to talk to me, she talks. When she does not, she spends two or three days without talking to me! That is really sad. I feel totally alone at home. She does things like that when I do not do something in the way she asks. It is like a punishment. She takes my money and does not give me a penny.. I do not eat well at home; she does not buy clothes, shoes, anything for me! When I ask for money, my money, she says: “I do not know what you want money for”. (I#3)*

As the quotations demonstrate, our participants are not only victims of psychological domestic violence. We can state that this type of abuse has co-occurred with verbal and financial abuse, and with neglect as well. We can not assume that the psychological domestic violence has increased women’s risk of suffering with other types of abuse.

#### Subtheme 1.2: The impact of psychological violence

As a result of psychological violence, the participants expressed feelings of sadness, hurt, anger, grief, and fear. Some of these feelings are evident in the following excerpts:

*It’s desperation! I have suffered a lot! I was afraid of her! And it is the same fear I had of my father. She hurts me so much with words. I feel so sad, so sad. You can never imagine what I have being through. (I#2)*

The participants also associated their violent situations to the physical and mental health symptoms:

*I have a problem with my stomach. I did five tests and nothing was found! It is the anger I swallow. I have this pain in my stomach because the anger I feel of him. (I#1)*

#### Subtheme 1.3: Responses of the older women to violence

Having, or not having, support helped to determine the responses of the participants to violence. They identified key individuals in their lives who had helped them in various ways.

*My oldest son spent all efforts to take me to live with him, but I do not want to leave my house. When I am afraid at home, I call my oldest son. He comes here really fast. I have two phones at home, one in the main room and other in my bedroom, so I can call my oldest son always when he [her youngest son] gets home drunk and violent. (I#8)*

However, not all participants had received the support they needed:

*I have talked to them. I have asked them to help me. The neighbours know everything, but they keep quiet! They do not want to get involved. No one comes here. No one! Only you came here today [crying]. (I#4)*

Despite the participants’ pain and suffering, some of the older women expressed feelings of compassion and protection for the abuser by denying/hiding the situation, withdrawing the complaint, or taking care of the abuser.

*My other children are angry. You heard him saying that I deny things that happen at home and that is true. I deny because I do not want a fight between my children. So, I hide it. I hide in order to the situation does not get worse. To avoid conflict, I call and ask for a car to take me to my other son’s house or to the farm, but always leave food, meat in the freezer, leave everything, to never let him starving. (I#5)*

Although hesitant, calling the police was a last resort as highlighted in the report bellow:

*I asked her to leave, but she did not go! I called the police because I was afraid that the situation was getting worse! She was very angry! Then the police talked to her and she came back home a little better, but soon returned to do what she was doing! When she moved, I was relieved. (I#2)*

According to the participants, police action worked initially but, for two of the participants, the situation was permanently solved only when the abuser left the older woman’s home. In all other cases, the abuser continued to act violently after returning from jail. After several failed attempts to address the violence, the participants who tried to face the situation (removing the abuser, leaving the house for a while, and reporting the situation) felt powerless, and decided to remain silent during the violence to protect themselves:

*Now I do not say anything, I do not give her attention, because if I say one thing, she says two and begins to fight. I prefer to remain silent! [Pause] I follow that old says: “One does not fight alone”. (I#5)*

### Theme 2: Expectations of the older women

This second theme represents the wishes of the participants regarding to the violent situation. The wishes are divided into two subthemes: to change the abuser’s behaviour or to leave the violent situation.

#### Subtheme 2.1: To change the abuser’s behaviour

Most of the participants loved their abusive family member and suffered from seeing them in prison or other difficult situations, so their greatest desire and expectation was to change the behaviour of the aggressor:

*I wanted a good person to talk to them about this, to advise them because I wanted them to know that it is wrong: if they want to live with me, they should talk with me properly (without violence), treat me well, know to appreciate the value of a mother. I want them to change, to improve in life, to be better persons, and then, we can live together again. (I#10)*

#### Subtheme 2.2: To leave the violent situation

Regardless of the actions taken by the participants, they shared a common desire of leaving the violent situation or hoping that aggressor leaves their homes. Even though they may not have had concrete alternatives to change their reality, they wanted to separate themselves from the abuser.

Hoping to leave the abusers’ house:

*I wanted to live with my other daughter. Her house is not far. I’ve wanted to move in with her, but I do not know if I will get the chance to do that [crying]. I cannot decide anything yet. If someone got a place for me, I would walk away from here. She is graduating in nursing. I am waiting for her to finish it, and then, I will live with her. I hope to be alive when it happens. (I#1)*

Hoping that the abuser would leave their houses:

*Ah, my wish is that she would go away from here! She would find a place for herself and leave me alone because I’m not living in a peaceful environment. If she left here, it would be the greatest happiness of the world for me because I suffer too much. I just quietly take all of it. I’m dying. (I#4)*

### Typification

After analysing the themes, authors established the typical characteristics of this social group. Participants have been threatened, disrespected, neglected, financially abused, forced to do housework, and humiliated. They expressed feelings of sadness, anger, grief, and fear, which impacted negatively on their health. The participants’ attempts to change their situation were unsuccessful and, as a result, they felt helpless. Their expectations were related to changing the abuser’s behaviour and the possibility of running away from the violent situation.

## Discussion

Interviews revealed the relationships among older women participants and their abusers during everyday life. These relationships were shaped by manifestations of violence such as verbal abuse, abusive gestures, threats, humiliation, disrespect, control, neglect, and exploitation. This violence generated feelings of sadness, anger, fear, hurt, and suffering. These abusive acts and behaviours represent some of the examples identified by Bavel [1] that included teasing, verbal degradation, threats, humiliation, coercion, intimidation, disenfranchisement, name calling, accusations, general disrespect, yelling, blaming, criticising, and offensive comments, among others. Also, these manifestations limited the older women’s ability to make decisions, and reduced their confidence and self-esteem.

Our participants also referred a lack of freedom. Bavel [1] identified various actions that family members undertake to isolate older people from society, such as: leaving them alone at home, not letting them use the phone, and controlling their lives. Strong evidence indicates that social isolation and a lack of social relations contribute to violence against older people who are already being victimized [30]. Failure to provide adequate food, clothes, shelter, medical care, and hygiene are examples of neglect acts [3].

Another manifestation of violence was psychological pressure, which some participants referred as ‘mental torture.’ This psychological pressure can also result from other forms of violence, such as financial, physical, verbal, or from neglect [31]. Our analyses revealed that psychological and financial abuse occurred concomitantly, fact that was mentioned in almost all of the interviews. Financial victimization is usually connected to other forms of violence. The abuser is usually a family member and the victims may fail to realize that financial abuse is a form of violence that should be reported [32]. This kind of abuse includes exploitation and/or use of funds or resources that belong to the older person [3].

Finally, analyses revealed the presence of ageism, the stereotypical and often negative bias involving age groups, and in particular older adults. Ageism, as any kind of discrimination, can essentially be thought as the applying of collective age-based characteristics to an individual, regardless of their individual features [33]. The older women and their abusers experience the interpersonal communication and the intersubjectivity, influencing and being influenced, understanding and being understood, acting and reacting upon each other [28]. Family members acted violently against the older women who responded through several strategies that frequently were unsuccessful. Both actions and reactions of the violence were influenced by the situational context experienced for the participants. Perceptions of violence and how violence manifests are influenced for each individual’s ‘stock of knowledge’, which is related to her/his ‘biographical situation’ and to ‘natural attitude of the world of everyday life’ [28]. In other words, the victims’ stock of knowledge influenced their own actions.

The participants’ daily lives involved diverse and ambivalent feelings. They felt sad, angry, afraid, and not at peace. Simultaneously, the participants love their family members, feel compassion, try to protect the aggressor and justify their violent actions. A study conducted in Ireland reported that participants feel a natural parental instinct to protect their children and to maintain the belief that they are good people, no matter what they do. This study also found that a situation considered abusive by the societies may be considered ‘normal’ within a family core [31]. Schutz described this parental instinct as a ‘natural attitude’ [28] and he defined the set of reasons for a social action as the intentionality of a person toward the accomplishment of a purpose [27]. Analyses of the experience of psychological violence lived by the older women in the study revealed ‘reasons why’, such as: the manifestation of violence, feelings (psychological reactions) and health- related consequences, compassion for the aggressor, need for support, limited availability of choices, and powerlessness. ‘Reasons for’ (i.e., guiding their future actions) included changing the aggressor’s behaviour or leaving the situation.

The interviews revealed that exposure to psychological violence affected participants’ physical and mental health. This concurred with a previous study that reported that older women suffer physically after experiencing years of violence [34].

The participants expressed the need to get help from someone, or to identify someone who might support them in the future. However, according to O’Brien *et al.* (2011), participants believed that older people do not appreciate a neighbour’s intervention in the their personal life, and they also felt inappropriate to involve external agencies such as the police or social services in household matters [31].

One effective way to address cases of domestic violence involves specialized services comprised by various professionals who are trained to help older people experiencing domestic violence. Nurses have been increasingly seen as effective professionals in the fields of individual and collective care related to violence, and forensic nurses may work in primary, secondary, and tertiary care [35]. In this sense, few services have been organized in Brazil to identify and report violence against older adults. The complexity of this issue requires various actions to deal with the many demands involved in these types of abusive situations [36].

The study participants had tried to face their violent situations, but felt powerless due to their limited support network, and they generally did not seek external help (e.g., police or social workers). Other research has also revealed that older adults who experience violence usually stay quiet due to the fear of reprisal, to the misinformation about their rights, or to the lack of appropriate support [36].

### Limitations

The findings of this qualitative study are limited to the specific context where we conducted it, what limits the transferability of the findings. However, this paper has provided a detailed description of the context, which could help to illustrate what may be relevant/applicable to other contexts. Once the collected data are based on participants’ self-reports, they are subject to possible recall bias. The process of data collection was also challenging because some participants did not want to talk about their own experience and, so, they only discussed the topic in general terms, what reduced the sample size. The declination of some participants shows the highly sensitive and ethical nature of this topic.

## Conclusions

The research findings in this study are useful for health care professionals. Victimized older women may ignore their own safety in order to protect the aggressor abuser, and they often tend to desire for changing the abuser’s behaviour. Highlighting this finding is relevant because Brazil has no programs focused on aggressors rehabilitating. The legal system addresses violence by arresting the abusive family member, which may be against the victim’s will. The research findings indicate that having the violent abuser arrested is not an option for many older women, so it may be helpful to develop strategies focused on the abuser’s rehabilitation. If our participants had more resources and information about domestic violence, they may have different expectations about their situations. One efficient form of emotional support might be to create group activities related to this topic at various community centres/agencies, where the victims could share their experiences and develop strategies to deal with the violence together with other people.

Future research might help to clarify the context of violence from the perspective of the abusive family member; to identify educational intervention for abusers; and to work with nurses in practice settings to evaluate the effectiveness of education and other interventions targeting abusers. Other priorities also include developing support services for victims of violence (such as shelters), creating educational programs for abuser, providing legal, social, and health information about resources to address violence against older women, as well as providing social, financial, and emotional support to the victims of elder abuse or referring them to other professionals/institutions who/that provide such services.
